# Entropy Production During Star Formation: An Analytic Thermodynamic Framework from the Main Sequence to Compact Remnants

**DOI:** 10.3390/e28090991

**Published:** 2026-09-04

**Authors:** Javier Martín-Torres, María-Paz Zorzano

**Affiliations:** 1 Earth-Life Science Institute (ELSI), Institute of Science Tokyo, Meguro-ku, Tokyo 152-8550, Japan; 2 Instituto Andaluz de Ciencias de la Tierra (CSIC-UGR), 18100 Armilla, Spain; 3 School of Engineering, Physics and Mathematics, Northumbria University, Newcastle-upon-Tyne NE1 8ST, UK; 4 Centro de Astrobiología (CSIC-INTA), Torrejón de Ardoz, 28850 Madrid, Spain; zorzanomm@cab.inta-csic.es

**Keywords:** star formation, entropy, second law of thermodynamics, Sackur–Tetrode equation, stellar evolution, compact objects

## Abstract

The transformation of a diffuse molecular cloud into a star necessarily increases the entropy of the universe, chiefly through the radiation emitted as gravitational binding energy is released. We present a compact, fully closed-form thermodynamic model of this process: the Sackur–Tetrode equation gives the entropy of the initial cloud and, generously, of the stellar material itself, while the released gravitational potential energy is converted into a radiation-entropy term $S_{\mathrm{rad}}=\Delta E_{\mathrm{pot}}/\left(2T_{\mathrm{eff}}\right)$, the factor of one-half following from the virial theorem for a self-gravitating star in hydrostatic equilibrium. For a solar-type star we obtain $\Delta S≃1.9\times 10^{37}\;J\;K^{-1}$, consistent with independent literature estimates of stellar and interstellar entropy. Extending the calculation across the main sequence (O through M) gives $\Delta S\propto M^{0.71}$, rising from $1.2\times 10^{37}\;J\;K^{-1}$ for a $0.3\;M_{⊙}$ M dwarf to $2.0\times 10^{38}\;J\;K^{-1}$ for a $20\;M_{⊙}$ O star. We then map the full $\left(M,R,T_{\mathrm{eff}}\right)$ parameter space to locate the locus of ΔS=0—the formal boundary of thermodynamic feasibility for a single monolithic collapse—and show that every real main-sequence star lies deep in the entropy-producing region, with the boundary itself displaced to radii and masses far outside the stellar regime. Applying the same closed-form model to representative red giants, supergiants, white dwarfs and neutron stars (not as a model of their true formation, but as a diagnostic of how compactness controls radiative entropy production) shows that ΔS is set primarily by the compactness $GM^{2}/\left(R\;T_{\mathrm{eff}}\right)$ of the final configuration, so that degenerate remnants—if they were assembled by a single collapse from a diffuse cloud—would be substantially larger entropy sources than main-sequence stars, while extended giants are comparatively modest ones. The same closed-form machinery gives direct access to a full thermodynamic feasibility map, something that would otherwise require a large grid of numerical simulations to reconstruct, and we compare our results throughout with the current literature on stellar and cosmic entropy rather than with ad hoc benchmarks.

## 1. Introduction

A molecular cloud collapsing to form a star becomes, by any local measure, a more ordered object: a diffuse, cold, roughly uniform gas cloud is replaced by a compact, hot, centrally concentrated body. The second law of thermodynamics is not violated by this local decrease in disorder because the collapse radiates far more entropy into the surrounding medium than is lost from the collapsing gas itself [1,2,3]. This basic bookkeeping problem—how much entropy is produced, and by what channel—has a long history and remains an active one. Martyushev and Zubarev [4] used photometric data to estimate the present-day entropy production rate of main-sequence stars, subgiants, giants and supergiants. de Avellar et al. [5] tracked the specific entropy per baryon of matter through the full sequence cloud → main-sequence star → white dwarf or neutron star → black hole, showing that gravity itself becomes the dominant entropy reservoir at the compact-remnant stage. Very recently, Elbaz [6] revisited the entropy radiated by stars over their main-sequence lifetime using a photon-gas treatment of the emitted radiation, finding that stars of very different masses converge to a nearly universal specific entropy production—a striking regularity for a different phase of stellar life than the one considered here, and one we return to in Section 7. On the star-forming cloud itself, Keto et al. [7] identified a distinct, turbulent form of entropy—built from the velocity dispersion and size scale of supersonic turbulence rather than from thermodynamic phase-space volume—whose dissipation controls whether a cloud fragments into multiple stars. On galactic and cluster scales, entropy thresholds have also been invoked as regulators of when star formation can proceed at all [8]. Detailed numerical treatments—radiation-hydrodynamical simulations of cluster formation [9], or explicit thermodynamic bookkeeping of a collapsing turbulent cloud [10]—capture much more of the real physics (turbulence, magnetic fields, radiative transfer, fragmentation) than any closed-form estimate can, at the cost of losing the transparency and reproducibility of a fully analytic model.

A closed-form treatment complements these more detailed approaches. It can be evaluated everywhere in parameter space at once, makes explicit which physical quantity controls the outcome, and provides a transparent, independently reproducible baseline—the same role played historically by other simple analytic estimates in star formation and stellar physics, such as the Jeans length or the Chandrasekhar mass.

The aim of this paper is to give a single, internally consistent, closed-form thermodynamic estimate of ΔS that (i) reproduces the right order of magnitude for the Sun, checked against independent literature values; (ii) extends cleanly across the entire main sequence; (iii) identifies the boundary in $\left(M,R,T_{\mathrm{eff}}\right)$ space at which the model formally predicts ΔS=0, and shows where real stars sit relative to it and (iv) applies the same closed-form machinery to representative evolved and compact objects, showing that the result is governed almost entirely by their compactness—which clarifies why such a calculation is not, and should not be read as, a model of their real formation history, while giving a clean quantitative illustration of why compact remnants are such deep gravitational-energy reservoirs. Every equation used below is either a standard textbook result—the Sackur–Tetrode equation, the virial theorem, the gravitational self-energy of a uniform sphere—or an explicit, stated approximation; we introduce no new microphysics.

## 2. Formalism

We adopt a two-state bookkeeping model. The *initial state* is a spherical, cold, roughly solar-composition molecular cloud of mass *M*, radius $R_{\mathrm{cloud}}$ and temperature $T_{\mathrm{cloud}}$, whose entropy we compute as an ideal monatomic gas via the Sackur–Tetrode equation [11,12]:

(1) $$S_{\mathrm{cloud}}=Nk_{B}\left[\ln\left(\frac{V}{N}{\left(\frac{2\pi mk_{B}T_{\mathrm{cloud}}}{h^{2}}\right)}^{3/2}\right)+\frac{5}{2}\right],$$

where $N=M/m_{H}$ is the number of (hydrogen) particles, $V=\frac{4}{3}\pi R_{\mathrm{cloud}}^{3}$, $m≃m_{H}$, and $k_{B},h$ are the Boltzmann and Planck constants. The coefficient inside the thermal-wavelength term is 2π: the quantity $\lambda \left(T_{\mathrm{cloud}}\right)=h/\sqrt{2\pi mk_{B}T_{\mathrm{cloud}}}$ is the standard thermal de Broglie wavelength, and $S_{\mathrm{cloud}}=Nk_{B}\left[\ln\left(V/\left(N\lambda^{3}\right)\right)+5/2\right]$ is the standard form of the equation (see, e.g., [13]), also adopted in recent applications to stellar entropy budgets [6]. A 4π coefficient is only correct if the equation is instead parameterized by the internal energy $U=\frac{3}{2}Nk_{B}T_{\mathrm{cloud}}$ rather than by $T_{\mathrm{cloud}}$ directly; we note this explicitly since the two parameterizations are easily conflated in practice.

The *final state* is the star itself, at its main-sequence radius $R_{🟉}$ and effective temperature $T_{\mathrm{eff}}$, plus the radiation that carried away the released gravitational energy. Treating the star as a uniform-density sphere, the gravitational potential energy released on collapse from $R_{\mathrm{cloud}}\gg R_{🟉}$ to $R_{🟉}$ is

(2) $$\Delta E_{\mathrm{pot}}≃\frac{3}{5}\frac{GM^{2}}{R_{🟉}}.$$

Only half of this released energy is radiated away. This is not an independent assumption but a direct consequence of the virial theorem applied to a self-gravitating ideal-gas star in hydrostatic equilibrium: $2K+U_{\mathrm{grav}}=0$, so that the star’s internal (thermal) energy at the end of contraction is $K=\frac{1}{2}\left|U_{\mathrm{grav}}\right|=\frac{1}{2}\Delta E_{\mathrm{pot}}$, and energy conservation then requires the radiated energy to be the remaining half, $E_{\mathrm{rad}}=\Delta E_{\mathrm{pot}}-K=\frac{1}{2}\Delta E_{\mathrm{pot}}$. This is the standard basis of Kelvin–Helmholtz contraction theory and is derived in every modern stellar-structure textbook (e.g., [14], Ch. 3). If this energy is radiated at temperature $T_{\mathrm{eff}}$, it carries entropy

(3) $$S_{\mathrm{rad}}=\frac{\Delta E_{\mathrm{pot}}}{2\;T_{\mathrm{eff}}}.$$

We use the star’s final, main-sequence $T_{\mathrm{eff}}$ as a single representative temperature for the whole radiative history, rather than integrating over the star’s actual pre-main-sequence contraction track, along which the photospheric temperature evolves (typically starting cooler, on the Hayashi track, before settling onto the main sequence). Because entropy per unit radiated energy scales as 1/T, using the final (typically higher) $T_{\mathrm{eff}}$ for the entire radiated budget is, if anything, a conservative choice: energy radiated earlier, at lower temperature, would have carried more entropy per joule than our single-temperature estimate assigns it. We flag this explicitly as a simplification made in the interest of a closed-form result, in the same spirit as the other idealizations in this model.

The star’s own matter also has some entropy, $S_{🟉}$; rather than model this with an ad hoc “stellar core” construction, we bound it using the specific entropy of main-sequence stellar material determined independently by Basu and Lynden-Bell [15] from a full statistical-mechanical treatment of the relevant equations of state: 11–$21\;k_{B}$ per baryon. This is the entropy associated with the internal energy retained by the star (the other half of $\Delta E_{\mathrm{pot}}$ in the virial argument above); as shown in Section 3, it is a small correction to ΔS for main-sequence stars, though—as we discuss explicitly in Section 6—this is no longer true for all the objects considered later in the paper. We carry $S_{🟉}$ as an uncertainty band rather than a point estimate throughout. The total entropy change is then

(4) $$\Delta S=\left(S_{🟉}+S_{\mathrm{rad}}\right)-S_{\mathrm{cloud}}.$$

This is deliberately the simplest model consistent with the second law and with the virial theorem: it tracks only the dominant radiative channel and ignores rotation, magnetic fields, turbulence, the detailed radiative-transfer history of the collapse, and any entropy carried by outflows. Numerical radiation-hydrodynamical simulations [9] and explicit microphysical treatments of the collapsing cloud itself [10] capture these effects at the cost of a closed-form result; our purpose here is the complementary one of providing a transparent, reproducible, order-of-magnitude-correct baseline.

## 3. Worked Example: A Solar-Type Star

Take $M=1\;M_{⊙}=1.989\times 10^{30}\;\mathrm{kg}$, $R_{\mathrm{cloud}}=0.1$ light-year $\approx 9.46\times 10^{14}\;m$, $T_{\mathrm{cloud}}=10\;K$—values typical of a dense prestellar core [1,2]. The number of particles is $N=1.19\times 10^{57}$ and the cloud volume is $V=3.55\times 10^{45}\;m^{3}$. Equation (1) then gives

(5) $$S_{\mathrm{cloud}}≃6.6\times 10^{35}\;J\;K^{-1},$$

equivalent to a specific entropy of $≃40\;k_{B}$ per baryon. This sits comfortably inside the range independently reported for diffuse interstellar gas, 20–$143\;k_{B}$ per baryon [15,16], which is a useful consistency check that does not depend on any single competing point-estimate.

For the final state, with $R_{🟉}=R_{⊙}=6.957\times 10^{8}\;m$ and $T_{\mathrm{eff}}=5772\;K$ (the nominal solar value),

(6) $$\Delta E_{\mathrm{pot}}≃\frac{3}{5}\frac{GM^{2}}{R_{🟉}}≃2.3\times 10^{41}\;J,$$

(7) $$S_{\mathrm{rad}}=\frac{\Delta E_{\mathrm{pot}}}{2\;T_{\mathrm{eff}}}≃2.0\times 10^{37}\;J\;K^{-1}.$$

The stellar-matter term, using 11–$21\;k_{B}$ per baryon for $N=1.19\times 10^{57}$ baryons, is $S_{🟉}≃\left(1.8–3.4\right)\times 10^{35}\;J\;K^{-1}$—about 1.3% of $S_{\mathrm{rad}}$ at its midpoint, confirming that it is legitimately a small correction here (though, as discussed in Section 6, this is specific to compact, hot configurations and does not hold for all objects considered later). Combining,

(8) $$\Delta S=\left(S_{🟉}+S_{\mathrm{rad}}\right)-S_{\mathrm{cloud}}≃1.9\times 10^{37}\;J\;K^{-1},$$

i.e., radiative dissipation exceeds the initial cloud entropy by roughly a factor of 30, so that ΔS is, to good approximation, simply $S_{\mathrm{rad}}-S_{\mathrm{cloud}}$. This order of magnitude, $∼10^{37}\;J\;K^{-1}$, matches the entropy-production estimates for main-sequence stars in Martyushev and Zubarev [4] and is consistent with the specific-entropy accounting of de Avellar et al. [5].

### Physical Nature and Formation Efficiency of the Fiducial Cloud

Two aspects of the fiducial cloud used above, and throughout the paper, warrant explicit justification: what kind of object it physically represents, and what fraction of its mass is assumed to end up in the star.

**Classification.** At $R_{\mathrm{cloud}}=0.1$ ly (≈6300 AU, ≈0.031 pc) and $M=1\;M_{⊙}$, the implied number density is $n\approx 3.4\times 10^{5}\;{\mathrm{cm}}^{-3}$ (rising to ≈$6.7\times 10^{6}\;{\mathrm{cm}}^{-3}$ for the $20\;M_{⊙}$ case and falling to ≈$1.0\times 10^{5}\;{\mathrm{cm}}^{-3}$ for the $0.3\;M_{⊙}$ case). This places the fiducial cloud squarely in the regime of a *dense, prestellar molecular cloud core* rather than a diffuse cloud or an entire giant molecular cloud (GMC): observed dense cores are ≳0.1 pc in size with densities of at least $10^{4}$–$10^{6}\;{\mathrm{cm}}^{-3}$ [17,18], and it is at this scale, not the much larger and more diffuse GMC scale, that direct core-to-star collapse is the relevant picture.

**Formation efficiency.** The calculation as presented uses the same mass *M* for the cloud and the final star, i.e., it implicitly assumes a core-to-star mass conversion efficiency of $\epsilon \equiv M_{🟉}/M_{\mathrm{cloud}}=1$. This is a genuine simplification: observationally, the prestellar core mass function is found to map onto the stellar initial mass function with an efficiency of ϵ≈20–40%, not 100% [18,19], the remainder being returned to the surrounding medium primarily via protostellar outflows. We adopt ϵ=1 as the simplest limiting case and now test explicitly how much this matters. Repeating the solar-type calculation with $M_{\mathrm{cloud}}=M_{🟉}/\epsilon >M_{🟉}$ (so that $S_{\mathrm{cloud}}$ is evaluated for the larger, pre-outflow reservoir while $S_{🟉}$ and $S_{\mathrm{rad}}$ still use the actual stellar mass) gives $\Delta S=1.87\times 10^{37}$, $1.79\times 10^{37}$ and $1.68\times 10^{37}\;J\;K^{-1}$ for ϵ=0.5, 0.3 and 0.2, respectively, compared with $1.93\times 10^{37}\;J\;K^{-1}$ at ϵ=1. Across this entire realistic range, ΔS changes by less than 15% and remains strictly positive and of the same order of magnitude, because the extra cloud entropy from the larger reservoir is logarithmic in $M_{\mathrm{cloud}}$ (Equation (1)) while $S_{\mathrm{rad}}$, which dominates ΔS, does not depend on the assumed efficiency at all. We, therefore, treat ϵ=1 as a transparent, order-of-magnitude-safe idealization rather than a load-bearing assumption, and do not carry a separate efficiency parameter through the rest of the paper.

## 4. The Main Sequence

**Figure 1:**
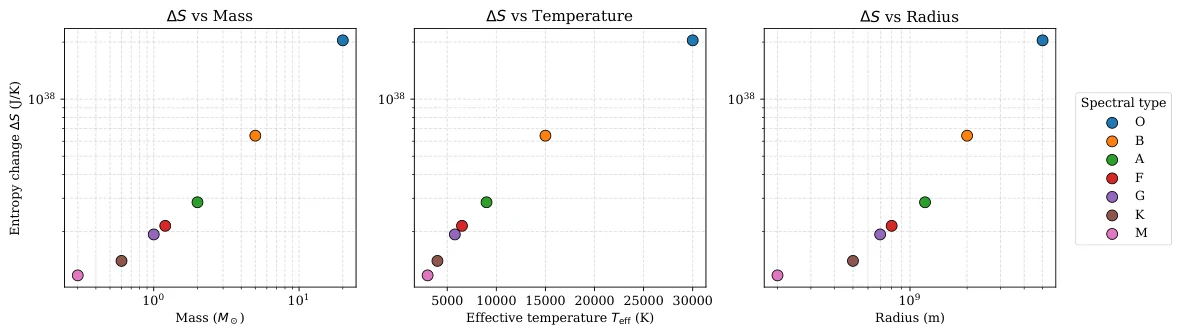
Entropy change ΔS across the main sequence as a function of stellar mass, effective temperature $T_{\mathrm{eff}}$, and radius (log–log for mass and radius; log-linear for temperature). Radiative dissipation drives a monotonic increase with all three parameters, since more massive, hotter, larger main-sequence stars release more gravitational binding energy overall even though $S_{\mathrm{rad}}\propto 1/\left(R_{🟉}T_{\mathrm{eff}}\right)$ individually disfavors large, cool stars.

Table 1 extends this calculation to representative O, B, A, F, G, K and M stars, using the same fiducial cloud ($R_{\mathrm{cloud}}=0.1$ ly, $T_{\mathrm{cloud}}=10$ K) and standard main-sequence radii and effective temperatures [20,21]. Figure 1 shows ΔS as a function of mass, radius and temperature. Radiative dissipation dominates at every mass, and ΔS increases monotonically with *M*, spanning $1.2\times 10^{37}\;J\;K^{-1}$ (M dwarf) to $2.0\times 10^{38}\;J\;K^{-1}$ (O star).

### A Self-Consistent Scaling Relation

The three free parameters (*M*, $R_{🟉}$, $T_{\mathrm{eff}}$) can be reduced to one using the empirical main-sequence mass–radius and mass–temperature relations. Fitting power laws directly to the seven $\left(M,R_{🟉},T_{\mathrm{eff}}\right)$ triples in Table 1 gives $R_{🟉}\propto M^{0.73}$ and $T_{\mathrm{eff}}\propto M^{0.56}$, in good agreement with the independently derived empirical relation of Demircan and Kahraman [21] ($R_{🟉}≃0.85\;{\left(M/M_{⊙}\right)}^{0.67}\;R_{⊙}$ for solar-type stars). We use these exact fitted exponents throughout, rather than rounding them to simple fractions, since rounding 0.56 to a “nice” value is easily conducted incorrectly (the nearest tenth is 0.6, not 0.5) and is in any case unnecessary once the exponents are already in hand from the fit. Substituting directly,

(9) $$S_{\mathrm{rad}}\left(M\right)=\frac{1}{2}\;\cdot \;\frac{3}{5}\frac{G\;M_{⊙}^{2-0.73-0.56}}{R_{0}T_{0}}\;M^{0.71},$$

where $R_{0}=6.39\times 10^{8}$ m and $T_{0}=5.80\times 10^{3}$ K are the fitted normalizations at $M=1\;M_{⊙}$; numerically, $S_{\mathrm{rad}}\left(M\right)≃2.14\times 10^{37}\;{\left(M/M_{⊙}\right)}^{0.71}\;J\;K^{-1}$. Because the exponent 0.71 is less than unity while $S_{\mathrm{cloud}}\left(M\right)$ grows only slightly slower than linearly in *M* (through the Nln(1/N)-type dependence of Equation (1) at fixed cloud size), $S_{\mathrm{cloud}}\left(M\right)$ eventually overtakes $S_{\mathrm{rad}}\left(M\right)$ at sufficiently large *M*: solving $S_{\mathrm{rad}}\left(M\right)=S_{\mathrm{cloud}}\left(M\right)$ numerically gives a crossing mass $M≃7.5\times 10^{5}\;M_{⊙}\;\left(1.5\times 10^{36}\;\mathrm{kg}\right)$—comparable to a globular cluster or the central regions of a small galaxy, and nearly four orders of magnitude beyond the most massive stars known (Section 5). We regard this purely as a demonstration that the scaling relation does not predict ΔS≤0 for any physically sensible stellar mass, not as a physical statement about star-cluster-scale objects, since the main-sequence mass–radius relation and the fixed-cloud-size assumption both lose meaning long before such masses are reached.

## 5. A Thermodynamic Feasibility Map

**Figure 2:**
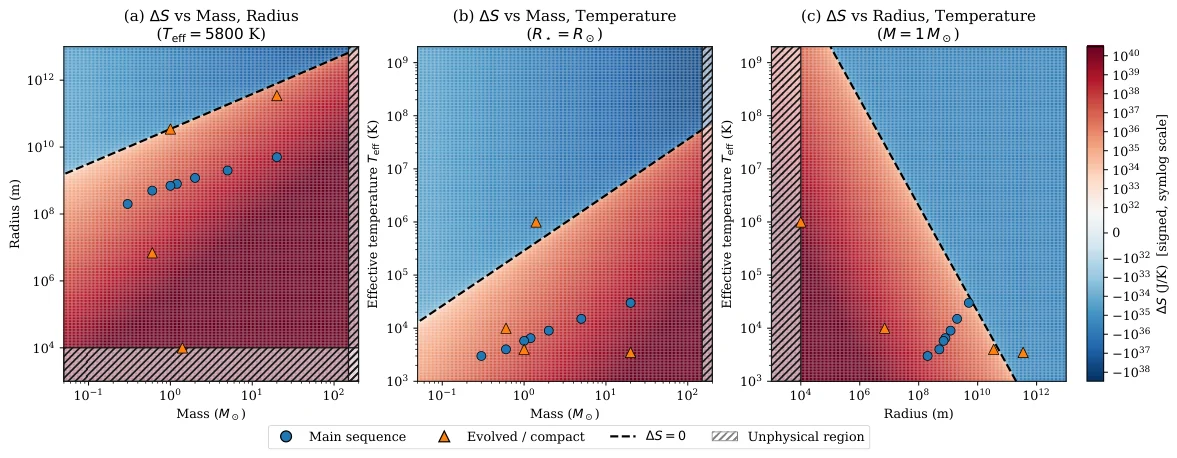
The thermodynamic feasibility map: ΔS (signed, symmetric-log color scale) as a function of two of $\left\{M,R_{🟉},T_{\mathrm{eff}}\right\}$ with the third held fixed, with the ΔS=0 boundary shown as a dashed line. Main-sequence stars (circles) and the four evolved/compact objects of Section 6 (triangles) are overlaid at their real $\left(M,R,T_{\mathrm{eff}}\right)$ values. Hatched, whitened bands mark parameter combinations outside any known stellar regime ($M>150\;M_{⊙}$; $R<10^{4}$ m), explained in the text. Every plotted object—main sequence *and* evolved—lies on the entropy-producing (ΔS>0) side of the boundary.

Because $\Delta S=S_{\mathrm{rad}}\left(M,R,T_{\mathrm{eff}}\right)-S_{\mathrm{cloud}}\left(M\right)$ (with $S_{🟉}$ included as in Equation (4)) is a closed-form function of three variables, it is straightforward to map out the full surface on which ΔS=0—the extremal boundary at which this toy model of direct monolithic collapse would produce *no* net entropy—and to ask where real objects sit relative to it. Figure 2 shows this for three orthogonal slices: $\Delta S(M,R_{🟉})$ at fixed $T_{\mathrm{eff}}=5800$ K; $\Delta S(M,T_{\mathrm{eff}})$ at fixed $R_{🟉}=R_{⊙}$ and $\Delta S(R_{🟉},T_{\mathrm{eff}})$ at fixed $M=1\;M_{⊙}$, with the ΔS=0 contour marked explicitly.

At fixed $T_{\mathrm{eff}}=5800$ K, the ΔS=0 boundary radius rises from $R_{\mathrm{boundary}}≃2.8\;R_{⊙}$ at $M=0.1\;M_{⊙}$ to ≃$650\;R_{⊙}$ at $M=20\;M_{⊙}$ (Table 2): in every case, dozens to hundreds of times larger than the actual main-sequence radius at that mass. Real main-sequence stars, therefore, sit deep inside the entropy-producing region, and would have to be inflated to giant- or supergiant-like radii, at fixed mass and effective temperature, before the model predicts ΔS≤0.

The two hatched bands in Figure 2 are not artefacts of the model but independent physical boundaries, included so that the map cannot be misread as extending to arbitrary $\left(M,R,T_{\mathrm{eff}}\right)$. The mass cut at $150\;M_{⊙}$ follows Figer [22], who found no stars above this mass in the Arches cluster despite a sample size for which ∼18 would be expected under an unbroken initial mass function, and concluded that stars simply do not form, or do not survive, much above this limit; subsequent analyses of individual very luminous stars in R136 have pushed plausible individual masses somewhat higher, but $150\;M_{⊙}$ remains the standard benchmark for “far beyond any ordinary star.” The radius cut at $10^{4}$ m follows from neutron stars being the most compact self-gravitating objects with a well-defined thermodynamic radius: precision NICER measurements give radii of 12–14 km for neutron stars near 1.4–$2.1\;M_{⊙}$ [23], so 10 km is already below any measured neutron-star radius. A stellar-mass object with $R<10^{4}$ m is, therefore, not merely rare but qualitatively different in kind: any further compaction pushes the object past what neutron degeneracy pressure can support, and it is no longer a star or stellar remnant with an ordinary thermodynamic entropy at all, but a black hole, whose entropy is governed by its horizon area rather than by any classical phase-space volume—a regime the Sackur–Tetrode formalism used throughout this paper simply does not describe.

## 6. Evolved and Compact Objects

We now apply the identical closed-form machinery—the same fiducial progenitor cloud, the same Equations (2)–(4)—to the present-day $\left(M,R,T_{\mathrm{eff}}\right)$ of a representative red giant, supergiant, white dwarf and neutron star (Table 3). We are explicit that this is a counterfactual calculation: none of these objects actually formed by direct collapse of a diffuse cloud to its present configuration, and for white dwarfs and neutron stars, the degenerate equation of state makes the classical Sackur–Tetrode formula for $S_{🟉}$ formally inapplicable (it can even return negative values in this regime, which is itself a signature of the classical formula’s breakdown, not a physical result). What the calculation does give, robustly, is the radiative-entropy consequence of a given final compactness $GM^{2}/\left(R\;T_{\mathrm{eff}}\right)$, which is a well-posed question even when the collapse history is not.

**Figure 3:**
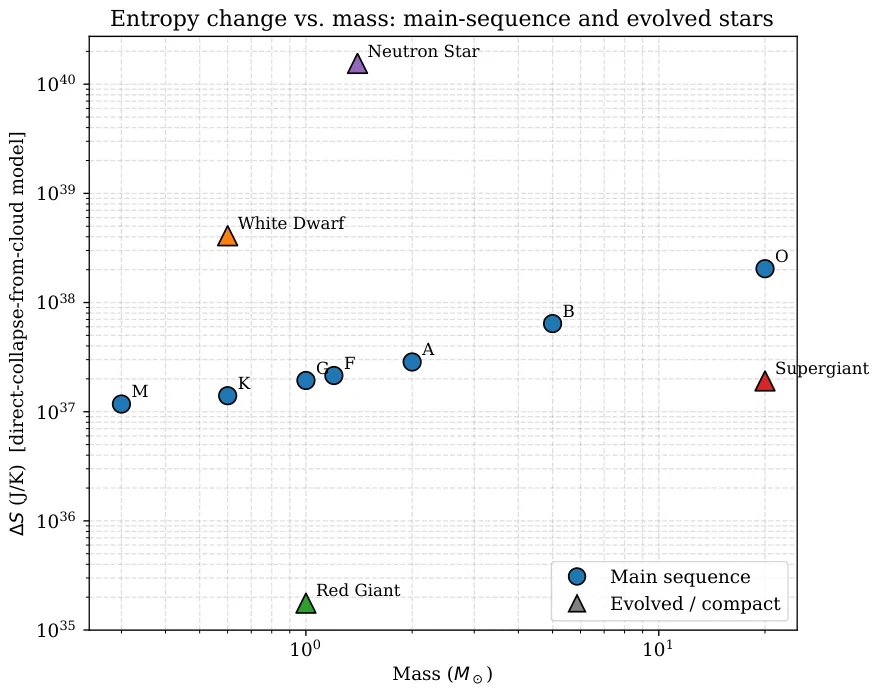
ΔS versus mass for main-sequence stars (circles) and the four evolved/compact objects of Table 3 (triangles), on the same fiducial-cloud convention throughout. All values are positive. Compactness, not evolutionary stage *per se*, controls the ordering: degenerate remnants (small *R*) lie far above the main sequence; extended giants (large *R*) lie below it.

Figure 3 places these alongside the main-sequence sequence of Table 1. Two features stand out. First, at fixed mass, the puffed-up, cooler evolved giants produce markedly less entropy in this model than their main-sequence counterparts of the same mass: the red giant’s ΔS is a factor of ∼110 below the $1\;M_{⊙}$ main-sequence value, and the supergiant’s is a factor of ∼11 below the $20\;M_{⊙}$ main-sequence value, because $S_{\mathrm{rad}}\propto R_{🟉}^{-1}$ penalizes their much larger radii more than their slightly lower temperatures compensate. Second, and in the opposite direction, the two degenerate remnants are substantial entropy sources in this counterfactual sense: at *R*∼$10^{4}$–$10^{7}$ m, their implied $\Delta E_{\mathrm{pot}}$ is so large that even at high $T_{\mathrm{eff}}$, $S_{\mathrm{rad}}$ exceeds every main-sequence value, by factors ranging from about 2 (white dwarf compared with the most massive O star) to over 1000 (neutron star compared with the least massive M dwarf)—roughly zero to just over three orders of magnitude, depending on which pair is compared. Encouragingly, the associated $\Delta E_{\mathrm{pot}}$ values (∼$8\times 10^{42}$ J for the white dwarf, ∼$3\times 10^{46}$ J for the neutron star—unaffected by the virial-theorem correction, which only concerns how this total is split between radiation and internal energy) are close to standard astrophysical estimates of the actual gravitational binding energies of such objects, which is a useful sanity check on the arithmetic even though, again, these objects did not form this way.

For the red giant specifically, we flag an important caveat that follows directly from the correction discussed in Section 2: because its large radius makes $S_{\mathrm{rad}}$ comparatively small, the stellar-matter term $S_{🟉}$—negligible for every main-sequence star and for the two degenerate remnants (≤2.5% and ≤0.04% of $S_{\mathrm{rad}}$, respectively)—amounts to 46% of $S_{\mathrm{rad}}$ for the red giant, and the reported ΔS, therefore, depends non-trivially on where within the Basu and Lynden-Bell [15] 11–$21\;k_{B}$-per-baryon range the true value lies (using the low end instead of the midpoint gives $\Delta S=0.9\times 10^{35}\;J\;K^{-1}$; the high end gives $2.6\times 10^{35}\;J\;K^{-1}$). The sign remains positive throughout this range, and the supergiant’s analogous sensitivity is much milder ($S_{🟉}\approx 20\%$ of $S_{\mathrm{rad}}$), but we consider it important to state plainly that the “$S_{🟉}$ is a small correction” simplification, safe for every other object in this paper, is the weakest link specifically for extended, low-compactness giants.

This picture is complementary to, and should not be confused with, the real physical trend established by de Avellar et al. [5]: tracking the true evolutionary history of a single star, the specific entropy per baryon decreases monotonically from the molecular-cloud stage through the main sequence to the white-dwarf or neutron-star stage, as gravity progressively confines the same matter into a smaller phase-space volume. Our calculation asks a different question: what if each final state, rather than the true progenitor, had been reached by one direct collapse from the fiducial cloud? The two pictures agree that compactness is the controlling variable. They necessarily disagree on whether the compact-object numbers should be read as “more” or “less” entropy than the main-sequence stage, since the two calculations are answering different questions despite sharing the same underlying variable, and this distinction is worth stating explicitly rather than leaving implicit.

## 7. Discussion

The closed-form approach adopted here earns its value beyond convenience. Because $\Delta S(M,R,T_{\mathrm{eff}})$ is available analytically, the full feasibility surface of Section 5 can be mapped directly, rather than reconstructed from a grid of simulations, and the outcome can be traced to a single combination, the compactness $GM^{2}/\left(RT_{\mathrm{eff}}\right)$ (Equations (2)–(4), with *G* the gravitational constant and *M*, *R*, $T_{\mathrm{eff}}$ the object’s mass, radius and effective temperature)—a dependence that a numerical scan would only reveal post hoc. This is the same role historically played by the Jeans length or the Chandrasekhar mass: neither was displaced by detailed numerical modeling, because their value was never numerical precision but transparency about why an effect occurs and at what scale.

The model reproduces the right order of magnitude for solar-type star formation without any free parameters beyond the fiducial cloud size and temperature, and its extension across the main sequence and to evolved objects is internally consistent once the virial-theorem factor of one-half is included in $S_{\mathrm{rad}}$ (Section 2). Its limitations are precisely those of any zero-dimensional, single-collapse thermodynamic estimate: it has no turbulence, no magnetic fields, no rotation, no fragmentation, no explicit radiative-transfer history, and—for degenerate matter—no valid microscopic entropy formula at all. Numerical radiation-hydrodynamical simulations [9] and dedicated treatments of cloud-collapse thermodynamics [10] are the appropriate tools when these effects matter quantitatively; the value of the present approach is its transparency and reproducibility as an order-of-magnitude baseline, in the same spirit as the specific-entropy-budget approach of Basu and Lynden-Bell [15], Egan and Lineweaver [16] and de Avellar et al. [5], with which our numbers are directly comparable. It is also a natural complement to the continuous entropy production during the main-sequence lifetime itself, which Elbaz [6] recently showed converges to a similar specific value across stellar masses using an independent, photon-counting approach; together, the two pictures suggest that stars are efficient entropy producers both in the single act of formation and continuously afterward.

A more fundamental limitation is worth stating explicitly rather than leaving implicit. We have used the standard, extensive Sackur–Tetrode formula throughout, for both the molecular cloud and (as a bound) the stellar material. This is the simplest and most transparent choice, but it is not obviously the correct one for a strongly self-gravitating system: because the gravitational interaction is long-range and unshielded, the additivity assumption underlying Boltzmann–Gibbs statistics—that the entropy of a composite system is the sum of its parts—need not hold, a point developed in detail in the classic review of Padmanabhan [24]. Nonextensive generalizations of statistical mechanics [25] have been explored as an alternative description of self-gravitating systems for exactly this reason, most often in the equilibrium, virialized context of dark-matter haloes: Lima and de Souza [26] and Leubner [27] derived power-law, polytrope-like density profiles from a nonextensive entropy functional, and Frigerio Martins et al. [28] fitted such profiles directly to a sample of spiral galaxy rotation curves, recovering an entropic index close to the standard Boltzmann–Gibbs value (q≃0.85) but statistically distinguishable from it. We stress that this remains a debated approach rather than a settled one: Féron and Hjorth [29] found that the simplest nonextensive prediction (a stellar-polytrope density profile) does not match simulated dark-matter haloes for any choice of polytropic index, which is a substantial objection to the simplest form of the theory. We have not attempted a nonextensive treatment of the cloud-collapse entropy here—the relevant regime for our problem (a transient collapse, far from virial equilibrium) differs from the equilibrium haloes this literature usually addresses, and adapting the formalism properly is a genuine calculation in its own right—but we flag the choice of extensive statistics as a real, unresolved simplification of this work rather than a settled one.

## 8. Conclusions

A closed-form thermodynamic model—Sackur–Tetrode entropy for a fiducial progenitor cloud, half the released gravitational binding energy (via the virial theorem) converted into radiated entropy—reproduces the correct order of magnitude, ΔS∼$2\times 10^{37}\;J\;K^{-1}$, for the formation of a solar-type star, and extends without modification across the main sequence ($\Delta S\propto M^{0.71}$, from $1.2\times 10^{37}$ to $2.0\times 10^{38}\;J\;K^{-1}$) and to representative evolved and compact objects. Mapping the full $\left(M,R,T_{\mathrm{eff}}\right)$ parameter space shows that real main-sequence stars lie far inside the entropy-producing region and that the formal ΔS=0 boundary sits many orders of magnitude away from any known star, whether approached at fixed temperature (a boundary radius of a few to several hundred solar radii) or via the reduced single-variable scaling relation (a crossing mass of order $10^{5}$–$10^{6}\;M_{⊙}$). Applied to evolved and compact objects as a diagnostic rather than a formation history, the same model shows that entropy production in this idealized sense tracks compactness, $GM^{2}/\left(RT_{\mathrm{eff}}\right)$, making degenerate remnants substantial entropy sources and extended giants comparatively modest ones—a finding that complements, rather than contradicts, the real evolutionary entropy trend established by de Avellar et al. [5] and the continuous main-sequence entropy production recently quantified by Elbaz [6]. We have also shown this picture to be robust: it is insensitive to the assumed core-to-star formation efficiency across the realistic observed range, and the fiducial cloud parameters correspond to a physically identifiable class of object (a dense prestellar core). A single closed-form expression is, therefore, sufficient to reproduce known benchmarks, extend them consistently across two orders of magnitude in stellar mass, and generate a full thermodynamic feasibility map that a comparable numerical study would need a large simulation grid to reconstruct—underscoring the continued value of transparent, analytic baselines alongside detailed numerical treatments of star formation and stellar evolution.

## Figures and Tables

**Table 1:** Entropy budget for main-sequence stars of each spectral type, computed from the same fiducial cloud. $S_{\mathrm{rad}}$ includes the virial-theorem factor of one-half (Equation (3)). The stellar term $S_{🟉}$ (11–21 $k_{B}$ per baryon) is folded into ΔS at its midpoint value.

Type	*M* ($M_{⊙}$)	$R_{🟉}$ (m)	$T_{\mathrm{eff}}$ (K)	$S_{\mathrm{cloud}}$ (J/K)	ΔS (J/K)
O	20	$5.00\times 10^{9}$	30,000	$1.21\times 10^{37}$	$2.04\times 10^{38}$
B	5	$2.00\times 10^{9}$	15,000	$3.14\times 10^{36}$	$6.42\times 10^{37}$
A	2	$1.20\times 10^{9}$	9000	$1.29\times 10^{36}$	$2.86\times 10^{37}$
F	1.2	$8.00\times 10^{8}$	6500	$7.83\times 10^{35}$	$2.15\times 10^{37}$
G	1.0	$6.96\times 10^{8}$	5772	$6.55\times 10^{35}$	$1.93\times 10^{37}$
K	0.6	$5.00\times 10^{8}$	4000	$3.98\times 10^{35}$	$1.40\times 10^{37}$
M	0.3	$2.00\times 10^{8}$	3000	$2.03\times 10^{35}$	$1.18\times 10^{37}$

**Table 2:** The ΔS=0 boundary radius at fixed $T_{\mathrm{eff}}=5800$ K, compared with the actual main-sequence radius at the same mass (from Table 1, interpolated where necessary).

*M* ($M_{⊙}$)	$R_{\mathrm{boundary}}$ ($R_{⊙}$)	Typical Actual $R_{🟉}$ ($R_{⊙}$)
0.1	2.83	∼0.15
0.3	8.73	0.29
1.0	29.96	1.0
5.0	156.11	2.9
20.0	647.88	7.2

**Table 3:** The same closed-form calculation applied to representative evolved/compact objects, using the identical fiducial cloud as Table 1. All four values are positive; the ordering is governed by compactness, not by evolutionary status. Unlike the main-sequence case, $S_{🟉}$ is not a negligible correction for the red giant and, to a lesser extent, the supergiant—see text.

Object	*M* ($M_{⊙}$)	*R* (m)	$T_{\mathrm{eff}}$ (K)	ΔS (J/K)
Red giant	1.0	$3.48\times 10^{10}$	4000	$1.76\times 10^{35}$
Supergiant	20.0	$3.48\times 10^{11}$	3500	$1.91\times 10^{37}$
White dwarf	0.6	$6.96\times 10^{6}$	10,000	$4.09\times 10^{38}$
Neutron star	1.4	$1.00\times 10^{4}$	$10^{6}$	$1.55\times 10^{40}$

## Data Availability

The Python (version 3.14.7) code that produces every number, table, and figure in this manuscript, together with the resulting data file, is provided as Supplementary Material.

## Supplementary Materials

The following supporting information can be downloaded at: https://www.mdpi.com/article/10.3390/e28090991/s1. File S1: verified_model. File S2: boundary_and_scaling. File S3: make_figures_1_and_2. File S4: make_figure3_feasibility_map.
